# Aldosterone and the Heart: Still an Unresolved Issue?

**DOI:** 10.3389/fendo.2014.00168

**Published:** 2014-10-14

**Authors:** Cristiana Catena, GianLuca Colussi, Francesca Nait, Flavia Martinis, Francesca Pezzutto, Leonardo A. Sechi

**Affiliations:** ^1^Hypertension Unit, Department of Experimental and Clinical Medical Sciences, Clinica Medica, University Hospital, University of Udine, Udine, Italy

**Keywords:** aldosterone, aldosterone-producing adenoma, bilateral adrenal hyperplasia, echocardiography, left ventricular hypertrophy, left ventricular mass, adrenalectomy, mineralocorticoid receptor antagonists

## Abstract

Receptors for mineralocorticoid hormones are expressed in myocardial cells and evidence obtained in animal studies suggests that activation of these receptors causes cardiac damage independent from blood pressure levels. In the last years, many of the issues related to the effects of aldosterone on the heart have received convincing answers and clinical investigation has focused on a variety of conditions including systolic and diastolic heart failure, arrhythmia, primary hypertension, and primary aldosteronism. Some issues, however, await clarification in order to obtain better understanding of what could be the role of aldosterone blockade in prevention and treatment of cardiovascular diseases. In this article, we overview the most recent findings of animal studies that have examined the contribution of aldosterone to cardiac function and clinical studies that have investigated the influence of aldosterone on left ventricular structure and function in the setting of primary hypertension and primary aldosteronism.

## Introduction

Aldosterone is an hormone secreted by the outermost portion of the adrenal cortex and participates to regulation of blood pressure by exerting its main effects on the distal nephron. At this site, aldosterone increases sodium and water reabsorption leading to expansion of the extracellular fluid volume. New experimental and clinical evidence that has emerged in the last decade indicates that, in addition to the actions on the kidney and contribution to body fluid and electrolyte balance, aldosterone affects many cell types where it regulates a variety of signal transduction mechanisms and cellular responses, the most relevant of which might result in tissue inflammation, hypertrophy, and fibrosis.

Expression of receptors for mineralocorticoid hormones (MR) has been detected in human cardiomyocytes and cardiac fibroblasts (1) and their protracted exposure to elevated circulating levels of aldosterone leads to myocardial damage that is unrelated to blood pressure changes (2). In fact, continuous infusion of aldosterone plus saline load in rodents causes inflammatory changes of the myocardium (3) that, in the long-term, result in tissue fibrosis (4). Also, and most important, myocardial fibrosis in these animal models of chronic aldosterone infusion is prevented by bilateral adrenalectomy or administration of MR blockers (5).

It is now more than a decade since two landmark clinical studies that investigated the effects of aldosterone antagonists in patients with advanced stages of cardiac insufficiency were published, reporting significantly decreased mortality with use of these drugs on top of standard treatment. The RALES study (6) was conducted in patients with New York Heart Association (NYHA) class III–IV heart failure who were treated with spironolactone and the EPHESUS study (7) in patients with myocardial infarction associated with severe left ventricular dysfunction who were treated with eplerenone, a MR antagonist without cross-reactivity with the receptor for androgens and progesterone. Later on, evidence of beneficial effects of aldosterone antagonists was extended to patients with earlier stages of heart failure in the EMPHASIS-HF study (8). In this trial, patients with NYHA class II cardiac failure and left ventricular ejection fraction of 35% or less received up to 50 mg/day of eplerenone in addition to standard treatment. The study was interrupted after a follow-up of <2 years because the composite cardiovascular outcome (cardiovascular death and hospitalization for heart failure) was 37% less frequent in patients who were treated with eplerenone than in those who had placebo. Taken together, these studies on systolic heart failure conveyed the information that blockade of the effects of aldosterone acts beneficially on the heart and provided convincing clinical evidence of the untoward effects of the hormone. Recent observations obtained in rodents models indicate that the cardiotoxic effects of aldosterone are mediated by oxidative activation of multifunctional Ca(2+)/calmodulin-dependent protein kinase II causing cardiac rupture and increased mortality after myocardial infarction (9).

Some issues related to the cardiac effects of aldosterone have found appropriate explanations during the last two decades. It has become clear that inappropriately high-aldosterone levels could induce myocardial damage and investigation on the interaction between aldosterone and the cardiovascular system has been expanded beyond systolic heart failure to other clinical conditions such as diastolic heart failure, arrhythmia, primary hypertension, and primary aldosteronism. Other issues, however, need better definition and the possible role of aldosterone blockade in prevention and treatment of cardiac conditions needs further investigation. This review summarizes the latest findings obtained in preclinical animal and human studies that have examined the impact of circulating aldosterone and MR activation on cardiac structure and function in hypertension.

## Aldosterone and the Heart: Animal Studies

As stated above, high-affinity MR have been detected in cardiac myocytes and fibroblasts (1) and their activation can induce myocardial damage with mechanisms that are independent of blood pressure elevation (2). Landmark experiments demonstrated that chronic aldosterone infusion caused fibrosis in the heart of rats that were fed a high-salt containing diet (4). In a subsequent series of elegant experiments, Rocha et al. demonstrated that inflammation of the perivascular tissue is antecedent to aldosterone-induced myocardial fibrosis (3) and that both inflammation and fibrosis can be effectively prevented by removal of the adrenal glands or, alternatively, by administration of aldosterone antagonists (5). Inappropriate elevation of aldosterone has been shown to lead to development of myocardial changes and the related cardiac dysfunction through a variety of mechanisms, including activation of oxidative stress (10, 11). In agreement with these observations, the beneficial effects on myocardial fibrosis of aldosterone antagonists (12) have been associated with a reduction of oxidative stress at the level of cardiomyocytes (13) and further evidence has been obtained in experiments conducted in genetically manipulated models.

Ventricular remodeling was investigated in mice with specific inactivation of cardiomyocyte MR [MR(MLCCre)] after an experimentally induced myocardial infarction (14). In these mice, cardiac structure and function were assessed 8 weeks after ligation of a coronary artery with evidence that ventricular dilatation and systolic dysfunction were significantly attenuated as compared to control mice. Preservation of ventricular volume and systolic function in mice with inactivated MR was associated with decreased generation of reactive oxygen species and better neovascularization of the infarcted area. In another study on a murine transgenic model [(*mRen2*)27 rat] characterized by persistent increase of tissue angiotensin-II and circulating aldosterone levels (15), it was reported that blockade of MR improves cardiac function. These transgenic animals had increased blood pressure and cardiac abnormalities consisting of impaired diastolic relaxation associated with ventricular hypertrophy and fibrosis and were treated with either hypotensive or non-hypotensive doses of spironolactone for 3 weeks that were compared with placebo. Non-invasive examination of the hearts demonstrated that both doses of spironolactone improved left ventricular diastolic function. Also, rats that were treated with placebo had evidence of increased generation of reactive oxygen species and increased biochemical markers of fibrosis that was not present in rats that received both doses of aldosterone antagonist. In summary, available evidence obtained in animal models with use of aldosterone antagonists or genetic manipulation of the MR indicates that aldosterone and its receptors contribute to both systolic and diastolic myocardial dysfunction with mechanisms that are tightly related to induction of oxidative stress. In addition to its impact on myocardial structure and function, aldosterone induces electrophysiological rearrangement of cardiomyocytes that are caused by changes in sarcolemmal ion transport systems and might explain the proarrhythmic effects of the hormone (16, 17).

In addition to direct effects on the myocardium, untoward effects of aldosterone on the heart could result from actions of the hormone on arterial vessels and, in particular, on the coronary arteries. Excess hypertrophy (18) and fibrosis (19) of the aorta were reported in hypertensive rat models with high-circulating aldosterone levels and it was demonstrated that vascular remodeling and fibrosis of hypertensive animals could be corrected by administration of eplerenone (20). Also, and most important, expression of MR was detected in vascular smooth muscle and endothelial cells obtained from human coronary arteries (21), suggesting that aldosterone might be involved in regulation of coronary flow. In support of this possibility, recent studies have reported the involvement of aldosterone and the MR in regulation of vascular tone. First, increased vascular response to a variety of vasoconstrictors has been demonstrated in genetically manipulated mice that over express the MR in endothelial cells (22). Second, aldosterone decreases the activity of the BKCa potassium channel that is associated with coronary smooth muscle cells (23) and is involved in regulation of coronary functional reserve. Third, investigation of the endothelium-dependent coronary responses in genetically manipulated mice that over express the MR has shown that acetylcholine-induced vasodilation of coronary arteries is significantly impaired (24). Of great relevance is the fact that in these mice impaired endothelium-dependent coronary vasodilation was prevented by treatment with either aldosterone antagonists or antioxidants, once again suggesting that increased generation of reactive oxygen species mediates the effects of aldosterone and MR activation on the heart and vessels.

## Aldosterone and Left Ventricular Changes in Primary Hypertension

Substantial clinical evidence indicates that left ventricular hypertrophy is associated with increased cardiovascular risk in hypertensive patients (25) and that antihypertensive treatments that lead to regression of hypertrophy decrease such risks (26). Therefore, it is important to understand whether aldosterone and MR-mediated mechanisms could contribute to ventricular hypertrophy and the related cardiac functional changes in primary hypertension. Many clinical studies reported a specific ability of renin–angiotensin system (RAS) blockers in causing regression of hypertension-induced left ventricular hypertrophy (27, 28). However, a significant proportion of patients with high-blood pressure and ventricular hypertrophy who are treated with either angiotensin-converting enzyme inhibitors or angiotensin-receptor blockers do not have their ventricular mass reduced by treatment. This might be explained, at least in part, by an “escape” of aldosterone production from the inhibitory effect of these drugs (29) and this possibility received support from a study that reported that left ventricular mass decreases with use of RAS blockers only in those hypertensive patients who had persistent decrease in plasma aldosterone levels (30). In agreement with this observation, two studies (31, 32) that were conducted in hypertensive patients with left ventricular hypertrophy showed that addition of MR antagonists to RAS blockers increased significantly the extent of ventricular mass reduction obtained with treatment, despite no significant difference was observed in blood pressure changes. The 4-E study was designed to compare the effects of enalapril, eplerenone, or the combination of the two drugs on hypertension-related left ventricular hypertrophy (33). After 9 months, decrease of ventricular mass was comparable in patients treated with enalapril or eplerenone, and the combination of the two drugs had significantly greater effects on left ventricular mass reduction than those obtained with each single drug. Another study conducted in hypertensive patients with left ventricular diastolic dysfunction demonstrated that low-dose spironolactone (25 mg/day) was more effective than placebo in restoring normal diastolic function (34). Finally, in a study of hypertensive patients who were unresponsive to the combination of three antihypertensive drugs, spironolactone (25–50 mg/day) was added to the ongoing treatment (35). In this study, spironolactone decreased significantly blood pressure and left ventricular mass with an extent of ventricular mass reduction that tended to be greater in patients with elevated pre-treatment plasma aldosterone levels.

As stated above, knowledge of possible contribution of aldosterone and MR-mediated mechanisms to the development and progression of hypertensive ventricular hypertrophy is relevant because hypertrophy is an important predictor of major cardiovascular events. Current evidence was principally obtained with use of MR antagonists and strongly supports a role for aldosterone as a determinant of left ventricular hypertrophy and diastolic dysfunction in primary hypertension, with important implications for treatment of these conditions. It is also important to notice that beneficial effects of MR antagonists on left ventricular structure and function could be relevant not only in patients with elevated plasma aldosterone but also in patients without evidence of aldosterone excess (36).

## Aldosterone and Left Ventricular Changes in Primary Aldosteronism

Primary aldosteronism is an endocrine disorder that is associated with hypertension, hypokalemia, metabolic alkalosis, suppressed plasma renin, and inappropriately elevated aldosterone secretion that is caused by an adrenal adenoma or bilateral adrenal hyperplasia. Early descriptions of patients with primary aldosteronism reported a low incidence of cardiovascular complications (37) that was generally explained by suppression of circulating angiotensin-II as a result of extracellular fluid volume expansion (38). More recent views, however, clearly indicate that long-term exposure to elevated circulating aldosterone results in substantial cardiac damage that occurs independent of the blood pressure levels (39, 40). Primary aldosteronism offers a unique clinical setting to investigate the effects of aldosterone on target organs because, in this condition, the effects of aldosterone are isolated from those of the RAS.

It is well known that abnormalities of the heart are common sequelae of hypertensive states and have been detected also in patients with primary aldosteronism. In fact, the majority of cardiac ultrasound evaluations conducted in patients with primary aldosteronism have found significantly greater left ventricular mass than in other types of hypertension (40, 41). In primary aldosteronism, increased frequency of inappropriately elevated ventricular mass has been reported also in the absence of left ventricular hypertrophy (42), suggesting that protracted exposure to high-aldosterone levels increases the ventricular mass beyond what is needed to compensate for the hypertension-related cardiac load. While left ventricular systolic function in primary aldosteronism does not generally differ from other types of hypertensive disease, an abnormal pattern of left ventricular filling is commonly detected, indicating diastolic dysfunction (43, 44). Abnormal filling has been associated with abnormalities of ultrasound densitometric patterns of the left ventricle (43), suggesting tissue fibrosis.

In addition to findings of cross-sectional studies, substantial information on the effects of aldosterone on the heart of patients with primary aldosteronism could be obtained from investigations conducted after treatment of this endocrine disorder. Most of these studies were short-term echocardiographic evaluations of patients who underwent unilateral adrenalectomy that reported normalization of left ventricular mass and diastolic filling properties (43, 44). In a first prospective, long-term follow-up, echocardiographic study of patients with primary aldosteronism who were treated with either surgery or spironolactone, we found comparable reduction of left ventricular mass, although this change occurred more rapidly in adrenalectomized patients (45). Notably, the extent of change in ventricular mass obtained with both treatments was directly related with pre-treatment plasma aldosterone levels (41). Later on, other studies have retrospectively analyzed the long-term cardiac outcome of surgical and medical treatment of primary aldosteronism reporting conflicting results (46–48). In fact, while adrenalectomy was almost consistently found to rapidly decrease left ventricular mass, effects of treatment with MR antagonists were inconsistent. Among other factors, differences in findings might be related to different duration of follow-up, inasmuch as reduction of left ventricular mass might require longer time to occur in patients treated with MR antagonists than surgery. Thus, effects of medical treatment of primary aldosteronism on ventricular mass remain controversial and this issue will be worthy of further evaluation in cumulative analyses that could increase the statistical power of each of these studies taken separately. In a recent study, patients with an aldosterone-producing adenoma associated with somatic mutations in the KCNJ5 potassium channel have been compared with patients without such mutations (49). Despite blood pressure levels and need for antihypertensive medications were comparable between patients with and without the KCNJ5 mutation, the former group had significantly greater plasma aldosterone levels and left ventricular mass. Surgical removal of adenoma in these patients, however, induced significant and comparable decrease in ventricular mass in patients with and without the potassium channel mutation. This observation suggests that differences in mechanisms that underlie the development of adrenal adenoma might affect the degree of cardiac involvement in primary aldosteronism, but not the possible benefits of treatment.

In summary, the evidence supporting a role of aldosterone in the development of cardiac hypertrophy that overtakes that of high-blood pressure itself is strong. As shown in many studies (50–53), patients with primary aldosteronism have greater frequency of coronary heart and cerebrovascular disease, congestive heart failure, and atrial fibrillation than matched patients with primary hypertension. Due to the relevance of ventricular hypertrophy in the assessment of cardiovascular risk, its regression might result in substantial protection from cardiovascular events even in patients with primary aldosteronism. This issue will have to be further investigated in adequately designed protocols.

## Conclusion

Robust experimental and clinical evidence indicates that aldosterone can cause myocardial tissue damage, including hypertrophy and fibrosis over that induced by high-blood pressure itself. Increased generation of reactive oxygen species appears to have a major role in most of the consequences on the cardiovascular system of aldosterone elevation and MR activation. Both in primary hypertension and hyperaldosteronism, aldosterone contributes to development and progression of left ventricular hypertrophy and thereby to cardiovascular risk. Therefore, MR antagonists could be beneficial in hypertensive states in terms of reduction of left ventricular mass and improvement of cardiovascular outcome. Although the important progress witnessed during the last years has contributed substantially to clarify many issues related to the effects of aldosterone and the heart, many others still await satisfactory answers. Further studies will be needed to address these issues and to explore the potential benefits of aldosterone blockade on the cardiovascular system.

## Conflict of Interest Statement

The authors declare that the research was conducted in the absence of any commercial or financial relationships that could be construed as a potential conflict of interest.
